# Hesperidin-, Curcumin-, and Amphotericin B- Based Nano-Formulations as Potential Antibacterials

**DOI:** 10.3390/antibiotics11050696

**Published:** 2022-05-20

**Authors:** Noor Akbar, Muhammad Kawish, Naveed Ahmed Khan, Muhammad Raza Shah, Ahmad M. Alharbi, Hasan Alfahemi, Ruqaiyyah Siddiqui

**Affiliations:** 1 College of Arts and Sciences, American University of Sharjah, Sharjah 26666, United Arab Emirates; noormicrobiologist555@gmail.com (N.A.); rsiddiqui@aus.edu (R.S.); 2 International Centre for Chemical and Biological Sciences, H.E.J. Research Institute of Chemistry, University of Karachi, Karachi 75270, Pakistan; kawishiqbal02@gmail.com (M.K.); raza_shahm@yahoo.com (M.R.S.); 3 Department of Clinical Sciences, College of Medicine, University of Sharjah, Sharjah 27272, United Arab Emirates; 4 Department of Clinical Laboratory Sciences, College of Applied Medical Sciences, Taif University, Taif 26521, Saudi Arabia; a.alharbii@tu.edu.sa; 5 Department of Medical Microbiology, Faculty of Medicine, Al-Baha University, Al-Baha 65799, Saudi Arabia; halfahmi@bu.edu.sa

**Keywords:** infectious diseases, multidrug resistance, nanoparticles, antibacterial activity, cytotoxicity

## Abstract

To combat the public health threat posed by multiple-drug-resistant (MDR) pathogens, new drugs with novel chemistry and modes of action are needed. In this study, several drugs including Hesperidin (HES), curcumin (CUR), and Amphotericin B (AmpB) drug–nanoparticle formulations were tested for antibacterial strength against MDR Gram-positive bacteria, including *Bacillus cereus*, *Streptococcus pyogenes*, Methicillin-resistant *Staphylococcus aureus* (MRSA), and *Streptococcus pneumoniae*, and Gram-negative bacteria, including *Escherichia coli* K1, *Pseudomonas aeruginosa*, *Salmonella enterica,* and *Serratia marcescens*. Nanoparticles were synthesized and subjected to Atomic force microscopy, Fourier transform-infrared spectroscopy, and Zetasizer for their detailed characterization. Antibacterial assays were performed to determine their bactericidal efficacy. Lactate dehydrogenase (LDH) assays were carried out to measure drugs’ and drug–nanoparticles’ cytotoxic effects on human cells. Spherical NPs ranging from 153 to 300 nm were successfully synthesized. Results from antibacterial assays revealed that drugs and drug–nanoparticle formulations exerted bactericidal activity against MDR bacteria. Hesperidin alone failed to exhibit antibacterial effects but, upon conjugation with cinnamic-acid-based magnetic nanoparticle, exerted significant bactericidal activity against both the Gram-positive and Gram-negative isolates. AmpB-LBA-MNPs produced consistent, potent antibacterial efficacy (100% kill) against all Gram-positive bacteria. AmpB-LBA-MNPs showed strong antibacterial activity against Gram-negative bacteria. Intriguingly, all the drugs and their conjugated counterpart except AmpB showed minimal cytotoxicity against human cells. In summary, these innovative nanoparticle formulations have the potential to be utilized as therapeutic agents against infections caused by MDR bacteria and represent a significant advancement in our effort to counter MDR bacterial infections.

## 1. Introduction

The introduction of antibiotics greatly reduced morbidity and mortality due to infectious diseases. However, the advent of multiple-drug-resistant (MDR) pathogenic microorganisms radically changed the scenario once again. Furthermore, MDR status is deteriorating [1,2]. Superbugs have emerged as a significant threat to current health care due to the scarcity of novel antimicrobial medications and the increased incidence of MDR bacteria that cause treatment failures [3]. It is a major public health concern, and it is critical to develop novel drugs or modify existing drugs to enhance their efficacy [4]. The ESKAPE pathogens (i.e., *Enterococcus faecium*, *Staphylococcus aureus*, *Klebsiella pneumoniae*, *Acinetobacter baumannii*, *Pseudomonas aeruginosa,* and *Enterobacter* spp.) have been highlighted as most emergent superbugs necessitating particular consideration because they are responsible for a number of nosocomial infections each year and have high antimicrobial resistance rates. With the discovery of Gram-negative ESKAPE bacterial isolates with diverse mechanisms of carbapenem resistance, the antibiotic of last option used to combat such infections, the demand for new antibiotic classes with novel modes of action is higher than ever. Unfortunately, just two new antibiotic classes have been introduced since the 1960s, and we are unable to keep pace with the emerging resistance [5]. In this scenario, it is imperative to search for groundbreaking antibacterial compounds that could fight this exacerbated antimicrobial resistance in superbugs or modify existing drugs to increase their efficacy. 

Nanotechnology based on nanomaterials has been used widely in health care settings, particularly as a new approach for infectious diseases [6]. Nanoparticles fight resistance in bacteria through a variety of processes. First, some nanoparticles, such as nitric oxide-releasing nanoparticles (NO NPs), chitosan-containing nanoparticles (chitosan NPs), and metal-containing nanoparticles, limit the development of resistance by employing various ways of attacking microbes simultaneously [7,8,9,10,11]. Another method of reducing resistance is to combine many antimicrobial agents into a composite nanoparticle [7,12]. Nanoparticles have also been employed to circumvent existing resistance mechanisms such as bacterial cell uptake and efflux of drugs, biofilm formation, and intracellular bacteria [7,9,12,13,14]. Finally, nanoparticles have been utilized to direct antibacterial drugs to the infection site, such as our group synthesized cinnamic-acid-coated iron oxide nanoparticles loaded with cefixime, showing potent bactericidal activity against Gram-positive and Gram-negative bacteria [15]. The significant enhancement in antimicrobial effect may be due to the functionalized cinnamic acid moiety [16], which possesses antimicrobial properties as well as iron oxide nanoparticles, which are well known for producing reactive oxygen species, so this effect, along with the drug, gives synergistic antimicrobial effect [17]. In another study by our group, lactoionic-acid-coated Zn-MOFs enhance the antibacterial efficacy of amoxicillin against *Helicobacter pylori* (Khan et al., 2021); the functionalized lactobionic acid moiety enhances the membrane permeability and allows for increased drug dosages to be delivered to the affected site, overcoming resistance with fewer side effects [6,18,19,20].

Hesperidin (HES) is a flavanone glycoside present in the orange peel that is utilized as a vascular-protecting compound alone or in combo to effectively combat several diseases [21]. In addition, HES presented notable antimicrobial, anti-inflammatory, antioxidant, and antitumor activities [22,23,24]. Despite promising therapeutic outcomes, poor aqueous solubility and the emergence of antimicrobial resistance limit the therapeutic efficacy of (HES) against bacterial isolates [25]. To address these shortcomings, various nanoformulations based on HES were designed; for instance, HES loaded into microemulsion exhibited remarkable antibacterial activity against several Gram-positive and Gram-negative bacteria [26]. Additionally, silver nanoparticles conjugated with HES present important antimicrobial activity against pathogenic bacteria and parasites [22]. Similarly, curcumin (CUR) exhibited strong antibacterial effects against several Gram-positive and Gram-negative pathogenic bacteria [27]. Curcumin-loaded nanovesicles showed promising antiparasitic activity against *Acanthamoeba castellanii* [28]. Cyclodextrin-loaded curcumin (CCD) presented potent antibacterial activity against *Escherichia coli* (*E. coli*) [29]. Considering the severe side effects, amphotericin B (AmpB) is an extensively prescribed antibiotic for treating systemic fungal infections [30,31]. AmpB is used in synergism with berberine to eliminate biofilms formed by *Candida albicans*/*S. aureus* [32]. A previous study revealed that AmpB forms ion channels in the bacterial plasma membrane; however, a significant dose of the drug is required [33]. In the present study, we synthesized HES-, CUR-, and AmpB-loaded magnetic nanoparticles and revealed their antibacterial efficiency against several MDR Gram-negative and Gram-positive bacteria, in contrast to the drugs alone. The outcomes of this study highlight the possibility of using nanoformulations to improve the clinical efficacy of currently used drugs in clinical settings. 

## 2. Materials and Methods

### 2.1. Materials

The purchased solvents are of High-Performance Liquid Chromatography (HPLC) grade and obtained from Fisher scientific, UK, through a local supplier. Dicyclohexyl carbodimide (DCC), 4-dimethyl aminopyridine (DMAP), ammonium hydroxide (NH_4_OH), cinnamic acid (CA), 3-aminopropyl silane (APT), ferrous sulfate heptahydrate (FeSO_4_·7H_2_O), ferric sulfate hexahydrate (Fe_2_(SO_4_)_3_·6H_2_O), lactobionic acid (LBA), Hesperidin (HES), Amphotericin B (AmpB), and curcumin (CUR) were purchased from Sigma Aldrich through a local supplier.

### 2.2. Preparation of CA-MNPs and HES-CA-MNPs Formulations 

The surface modification of magnetic nanoparticles (MNPs) with CA was performed in various steps. Firstly, MNPs were synthesized co-precipitation technique in accordance with previously published protocol [34]. The synthesized MNPs were then subjected to silane functionalization with APT, as previously reported [35]. CA coating at APT-MNPs was conducted as previously reported [34]. Briefly, CA (2.02 mmol) was added to a flask containing dimethyl formamide (DMF) along with DCC (2.42 mmol) and DMAP (0.081 mmol) with constant stirring for 10 min. Then, APT-MNPs (0.3 g) were added, and the reaction was progressed with constant stirring for 24 h. CA-MNPs were obtained via sequential washing with DMF and stored at 4 °C for further analysis. HES loading onto CA-MNPs was performed in accordance with our previously published protocol [15]. Concisely, CA-MNPs (1 mg/mL) were prepared and mixed with three different concentrations of HES (1–3 mg/mL) in separate flasks incubated at 200 rpm for 24 h to facilitate the drug uptake. After 24 h, the drug-loaded suspensions were centrifuged, and the obtained supernatant was analyzed at 261 nm on an ultraviolet–visible (UV-VIS) spectrophotometer. The suspension containing a higher amount of drug was selected for further analysis. 

### 2.3. Preparation of LBA-MNPs, CUR-LBA-MNPs, and AmpB-LBA-MNPs

The functionalization of LBA onto the surface of NPs was established by adopting a previously published protocol [35,36,37]. Briefly, LBA (1.76 mmol) was solubilized in DMF containing DMAP (0.081 mmol), followed by stirring for 10 min under inert atmosphere. Then, APT-MNPs (0.19 g) were added and then dropped by the addition of DCC (1.69 mmol); the reaction was allowed to progress for 24 h. The resultant surface-functionalized MNPs were washed with DMF and dried at −20 °C on a freeze dryer (Vritis 25 SRC, USA) overnight. CUR loading was performed in accordance with the previously reported protocol [38,39]. Briefly, different amounts of CUR (1 mg to 3 mg) were dissolved in methanol containing LBA-MNPs (1 mg/mL). The obtained CUR-LBA-MNPs were removed via centrifugation at 12,000 rpm, and the supernatant containing the unloaded drug was measured by UV-Visible spectrophotometer (Thermo Scientific Evolution 220, Shanghai, China) at λ = 425 nm. The ratio containing higher loading capacity and narrow size was selected for further analysis. LBA-MNPs were also exploited for their drug entrapment potential against AmpB using the passive drug-loading technique. Briefly, LBA-MNPs were incubated with various equivalents of AmpB in methanol for 24 h on a shaker at 200 rpm under ambient conditions. The resulting AmpB-LBA-MNPs were removed by means of a permanent magnet and washed sequentially with water to remove the unloaded drug, which was further analyzed on UV at λ = 405 nm, and the obtained CUR-LBA-MNPs and AmpB-LBA-MNPs were stored at 4 °C for further analysis. 

### 2.4. Hydrodynamic Diameter, Polydispersity Index (PDI), and Morphology

The average size and PDI and CA-MNPs, LBA-MNPs, HES-CA-MNPs, CUR-LBA-MNPs, and AmpB-LBA-MNPs were analyzed from Zetasizer (Zetasizer Nano ZS90 Malvern Instruments, Malvern, UK). Briefly, nanosuspensions were transferred to a plastic cuvette with caution to avoid air bubbles. The cuvette was then placed in a spectrometer, and the study was conducted at room temperature. The medium viscosity, pressure, and refractive index were set at 1.0, 80.4, and 1.33, respectively. CA-MNPs, LBA-MNPs, HES-CA-MNPs, CUR-LBA-MNPs, and AmpB-LBA-MNPs were further evaluated for surface morphological analysis using atomic force microscopy (AFM, Agilent 5500, Agilent, Santa Clara, CA, USA). The nano-suspension was placed as a drop on a mica slide, dried at room temperature, and then mounted on a microscope for imaging at non-contact mode.

### 2.5. Drug-Loading Efficiency Determination

The drug-loading efficiency of HES-CA-MNPs, CUR-LBA-MNPs, and AmpB-LBA-MNPs was studied by adapting protocol [40,41]. Briefly, the nano-suspensions were centrifuged at 12,000 rpm for 15 min to separate NPs. After successive dilution of the supernatant, it was analyzed at λ_max_ of drugs using a UV-VIS spectrophotometer. The percent entrapment efficiencies were calculated by using the following equation:

(1) $$\%\;\text{Drug Loading}=\frac{\text{Amount of drug used}\;-\mathrm{unloaded drug}\;}{\text{Amount of drug used}\;}\times 100$$

### 2.6. Bacteria Used in This Study

In the present study, numerous multidrug-resistant Gram-negative bacteria including *E. coli* K1, *Serratia marcescens*, *P. aeruginosa,* and *Salmonella enterica* as well as Gram-positive bacteria such *B. cereus*, *S. pneumoniae*, Methicillin-resistant *S. aureus* (MRSA), and *S. pyogenes* were used (Table 1). All these bacterial species were isolated from clinical samples. Prior to the experiments, these bacteria were cultured in nutrient broth (NB) overnight at 37 °C in aerobic conditions, as described before [15,42].

### 2.7. In Vitro Antibacterial Assays

Antibacterial assays were used to investigate the bactericidal properties of drugs, NPs alone, and nanoconjugates against MDR bacteria, as described previously [43,44]. Briefly, the optical density (O. D.) of overnight grown bacterial culture was adjusted to O. D. = 0.22 at λ = 595 nm using a spectrophotometer. Next, 1 × 10^6^ CFU/mL bacterial inoculum was treated with drugs, NPs, and drug–NP conjugates at 100 µg/mL for 2 h at 37 °C aerobically. After this incubation, pre-treated bacterial cultures were ten-fold serially diluted, and different dilutions (i.e., 10^−3^, 10^−4^, 10^−5^, and 10^−6^) were plated on nutrient agar plates. The plates were then incubated overnight at 37 °C, and bacterial colonies were counted to determine viable bacterial colony-forming units (CFUs/mL). Methanol (CH_3_OH) was used as solvent control since all the drugs and nanoformulations were dissolved in methanol. Bacteria incubated with phosphate-buffered saline (PBS) were used as negative control, while incubated with gentamicin was taken as positive control. 

### 2.8. Minimum Inhibitory Concentration

Using broth micro dilution assays, drugs, NPs, and drug–NP conjugates were examined to determine their minimum inhibitory concentration against *P. aeruginosa* and MRSA [43,45,46]. Drugs, NPs, and their nanoconjugates were two-fold serially diluted in Muller Hinton broth at concentrations ranging from 3.125 µg/mL to 200 µg/mL. After that, the bacterial O. D. was set equal to 0.5 McFarland’s standard. The growth and sterility controls were MHB alone and bacteria seeded in MHB, respectively. The plates were incubated at 37 °C for 24 h. The MIC endpoint is the concentration of drugs, NPs, and nanoconjugates at which no visible growth in the tubes occurs. The O. D. of the tubes was measured before and after incubation to confirm the MIC values. 

### 2.9. In Vitro Cell Cytotoxicity Assays

Host cell cytotoxicity assays were accomplished using Lactate dehydrogenase (LDH) assays as described earlier [47,48]. Briefly, drugs, NPs, and their nanoconjugates were incubated at 100 µg/mL with confluent human cells (HeLa ATCC^®^ CCL2™) monolayer in a 96-well plate for 24 h at 37 °C with 5% CO_2_ in humidified condition. Following this, 1% Triton X-100 was added to the positive control well and incubated on the plate for 60 min at 37 °C. Then, an equal amount of cells supernatant (comprising LDH enzyme) from each well was combined with an equal amount of LDH kit reagents, and cytotoxicity was determined in proportion to LDH released from cervical cancer cells using a spectrophotometer at 490 nm. The formula for percent cytotoxicity is as follows:

(2) $$\%\;\text{Cytotoxicity}=\frac{\text{Sample value}\;-\text{negative control value}\;}{\text{Positive control value}-\text{negative control value}}\times 100$$

For negative control, HeLa cells were grown in RPMI alone well having 1% Triton X-100 was taken as positive control.

### 2.10. In Vitro Release Study

The dialysis method was used to study the drug release profile of HES, CUR, and AmpB from nanostructures with slight modification to our previously published protocol [49]. Concisely, 5 mg of each HES-CA-MNPs, CUR-LBA-MNPs, and AmpB-LBA-MNPs were dissolved in buffers (4 mL; pH 4.0 and pH 7.4) having 0.1% SDS and loaded into dialysis bag. The bags were then placed in a flask containing 40 mL buffer (pH 4.0 and pH 7.4), followed by shaking at 100 rpm at 37 °C for 24 h. The samples (2 mL) were drawn from the flask at specific intervals and replaced by fresh buffer, and the acquired samples were quantified via UV-Vis spectrophotometer. 

### 2.11. Statistical Analysis

For every investigational study, three independent experiments were executed, and each condition was performed in duplicate. Data were expressed as means ± standard errors of the means (SEM), whereas representative results were selected. All data were analyzed for significance by Student T-test using (Graphpad Prism 8.0.2 Software San Diego, CA, USA). A *p*-value ≤ 0.05 was deemed significant.

## 3. Results

### 3.1. Preparation of CA-MNPs and HES-CA-MNPs Formulations

**Scheme 1:**
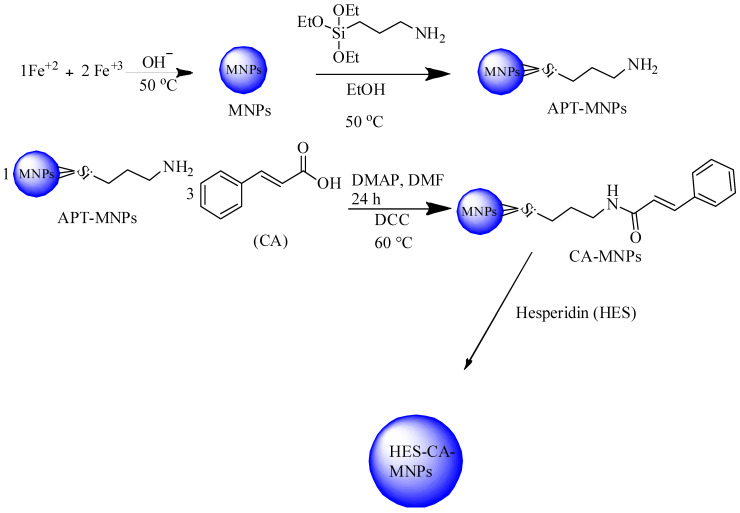
Synthetic scheme of CA-MNPs.

**Figure 1:**
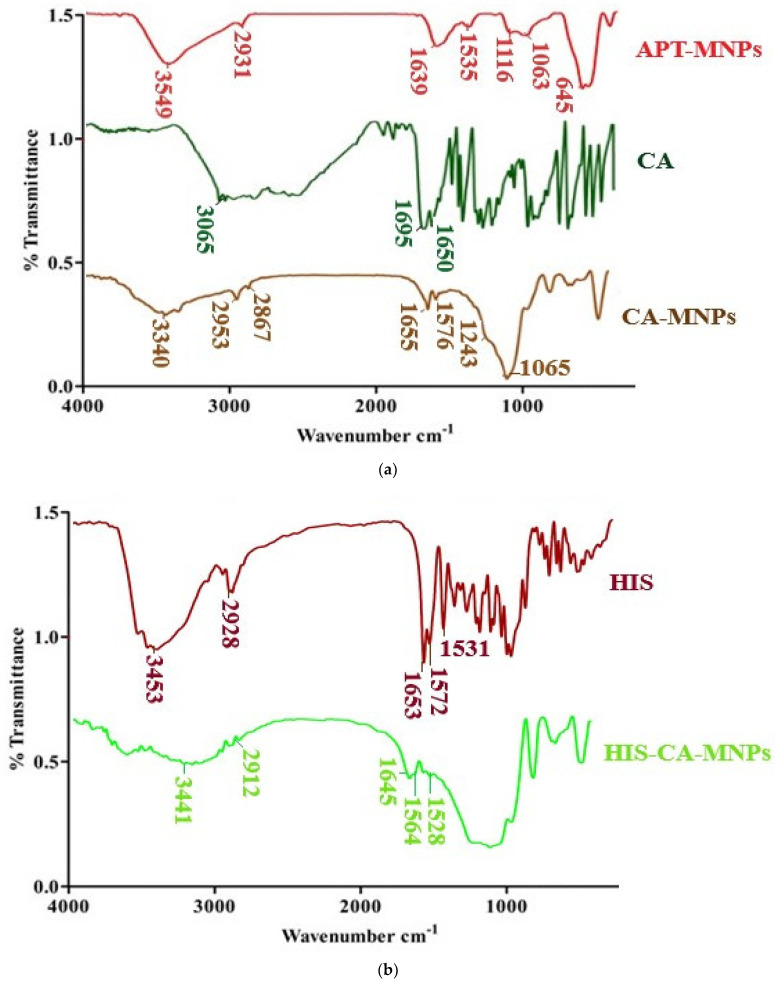
(**a**) FTIR spectra of APT-MNPs, CA, and surface-coated CA-MNPs. (**b**) FTIR spectra of HES along with HES-CA-MNPs.

The preparation of CA-MNPs is discussed in (Scheme 1), and Fourier transformed infrared (FTIR) spectra of APT-MNPs, CA, and CA-MNPs are presented in (Figure 1). The APT-MNPs show distinctive Si-O stretching bands at 1116 cm^−1^ and 1063 cm^−1^; in addition, NH_2_ bending and stretching vibrations around 3459 cm^−1^, 1639 cm^−1^, and 2941 cm^−1^, and 645 cm^−1^ of (C-H) and (Fe-O) vibrations was indicative for APT modification onto MNPs [50]. CA alone is showing broad absorption around 3065–2000 cm^−1^, indicating the presence of COOH moiety. Furthermore, (C=O) and (C=C) absorptions are also observed at 1695 and 1650 cm^−1^, as previously reported [51]. The COOH broad stretching band of CA diminishes when it is functionalized onto APT-MNPs (Figure 1a). The (N-H) stretching of amide was observed at 3340 cm^−1^ in combination with aliphatic and aromatic (C-H) at around 2953 cm^−1^ and 2867 cm^−1^, respectively. The (C=O) absorption of amide at 1655 cm^−1^ along with aromatic (C=C) stretch around 1576 cm^−1^ and (Si-O) stretch at 1243 cm^−1^ and 1065 cm^−1^ complies with the formation of CA-MNPs, as indicated by our previously published report [15,52]. FTIR spectra of HES show its characteristic absorption frequencies at 3455 cm^−1^ and 2944 cm^−1^ correspond to a hydroxyl group and stretching of –CH functional groups, respectively (Figure 1b). Similarly, the peak for C=O stretching appears at 1645 cm^−1^, while peaks for aromatic C=C appear at 1582 cm^−1^ and 1523 cm^−1^, as represented in (Figure 1b) [22,53]. FTIR spectrum of HES-CA-MNPs shows all the show slight variation in characteristic peaks of HES at 3441 cm^−1^, 2912 cm^−1^, and 1648 cm^−1^ of (N-H), (C-H), and (C=O) stretching vibrations. Moreover, a shift in the aromatic stretch was also observed at 1564 cm^−1^ and 1528 cm^−1^, which attributes that HIS is adsorbed onto the surface of CA-MNPs via noncovalent interaction of HES functional groups with CA-MNPs [54,55].

### 3.2. Preparation of LBA-MNPs, CUR-LBA-MNPs, and AmpB-LBA-MNPs

**Scheme 2:**
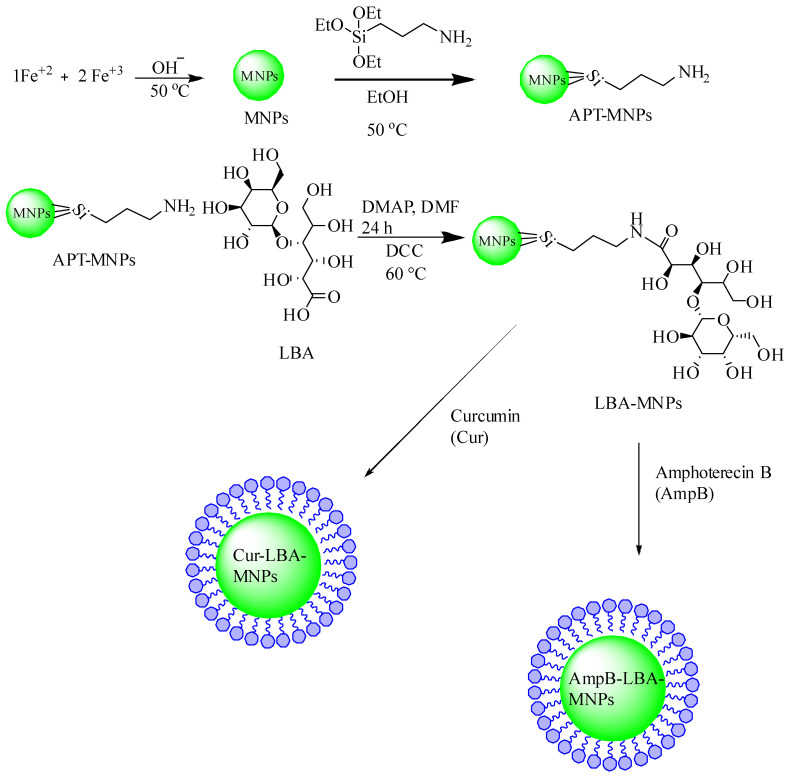
Synthetic scheme of LBA-MNPs.

**Figure 2:**
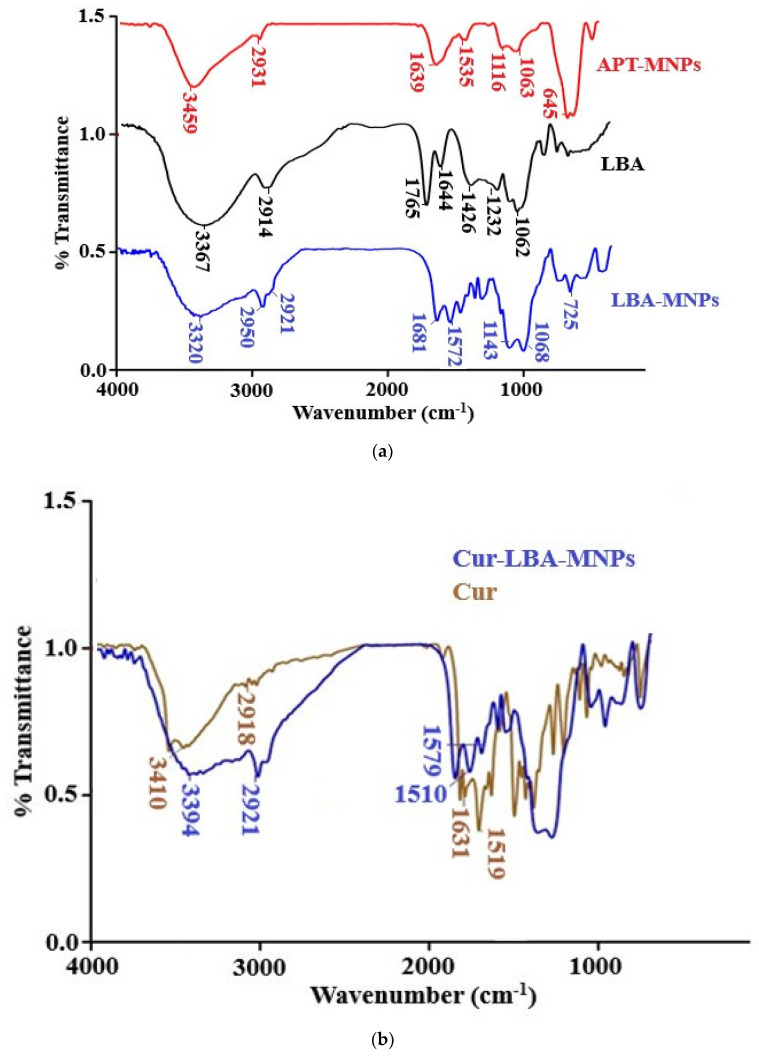

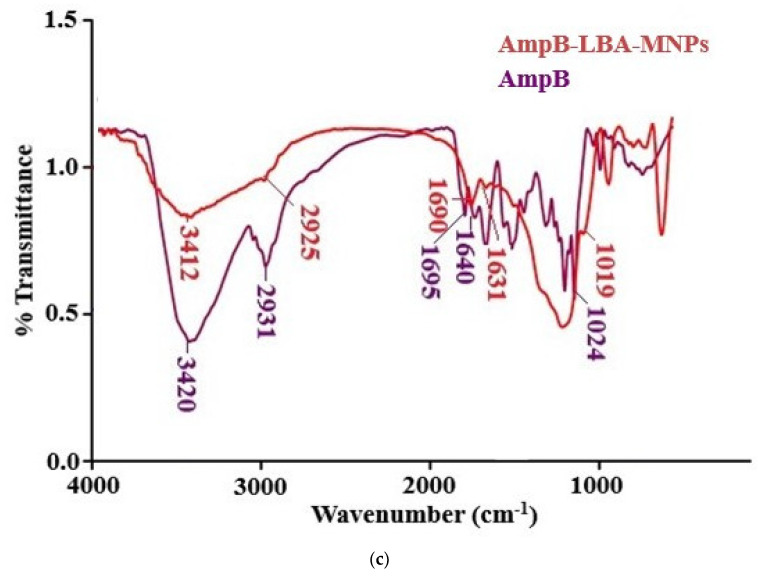
(**a**) FTIR spectra of APT-MNPs, LBA, and surface-functionalized LBA-MNPs; (**b**) FTIR spectra of CUR and CUR-LBA-MNPs; (**c**) FTIR spectra of AmpB and AmpB-LBA-MNPs.

The synthesis of amino-functionalized MNPs and their LBA conjugated analogs is shown in Scheme 2. FTIR spectra of surface fabrication of APT onto MNPs and their modification with LBA are shown in Figure 2a. The APT-MNPs show distinctive Si-O stretching bands at 1116 cm^−1^ and 1063 cm^−1^ in addition to NH_2_ bending and stretching vibrations around 3459 cm^−1^ and 1639 cm^−1^, which was in agreement with the previous reports [15,50]. The stretching frequency around 2931 cm^−1^ corresponds to the propyl group. When functionalized with LBA, the frequency around 1681 cm^−1^ and 1068 cm^−1^ corresponds to amide C=O and C-O-C, respectively, which validate the conjugation of LBA on MNPs as published in our previous report [34,36,56,57]. Additionally, increased absorption at 3320 cm^−1^ corresponds to the hydroxyl group of functionalized ligands [37]. These bands comply with the formation of LBA-MNPs [57]. The FTIR spectrum of CUR show characteristics peak at 3410 cm^−1^ for the OH stretching. Moreover, C-H, C=C, and C=O absorptions were also observed at 2918 cm^−1^, 1631 cm^−1^, and 1519 cm^−1^, respectively [58]. In drug-loaded CUR-LBA-MNPs formulation, slight variation was observed for OH stretching as the peak shifted at 3394 cm^−1^, while C=C and C=O stretching frequencies were shifted at 1579 cm^−1^ and 1510 cm^−1^. Figure 2b shows the adsorption of CUR onto the surface of LBA-MNPs [34,59].

AmpB reveals characteristic absorption around 1695 cm^−1^ and 1640 cm^−1^, which corresponds to (C=O) and (C=C) moiety [60]. The stretching frequency at 3420 cm^−1^ corresponds to OH stretching. AmpB-LBA-MNPs nanoparticles show slight variation in absorption frequencies; a peak at 1695 cm^−1^ of carboxylic acid (C=O) was shifted at 1690 cm^−1^, and a peak at 1640 cm^−1^ was shifted at 1631 cm^−1^. The peak at 1024 cm^−1^ of the acetal bond was shifted to 1019 cm^−1^ (Figure 2c). The absorption at 3420 cm^−1^ of OH was shifted to 3412 cm^−1^ (Figure 2c), which is attributed to the fact that AmpB was adsorbed onto the surface of LBA-MNPs via hydrogen bonding of hydroxyl groups with LBA and π−π stacking interaction between the drug and the synthesized NPs [49]. 

### 3.3. Hydrodynamic Diameter, Polydispersity Index (PDI), and Morphology 

**Figure 3:**
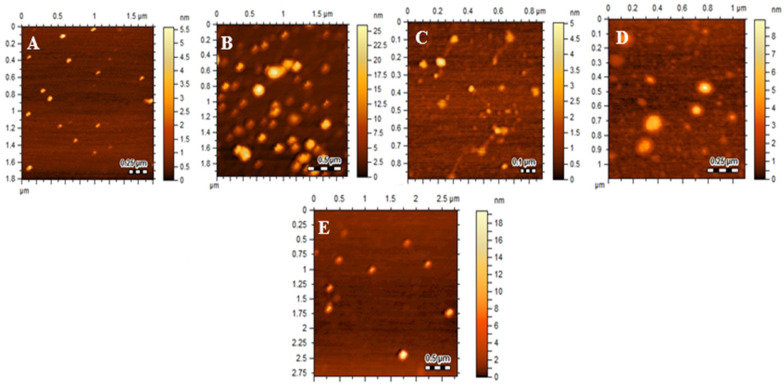
Atomic force microscopic images of (**A**) LBA-MNPs, (**B**) CA-MNPs, (**C**) HES-CA-MNPs, (**D**) CUR-LBA-MNPs, and (**E**) AmpB-CA-MNPs.

The average sizes of CA-MNPs, LBA-MNPs, HES-CA-MNPs, CUR-LBA-MNPs, and AmpB-LBA-MNPs are depicted in Table 2. The increment in size of CUR-LBA-MNPs and AmpB-LBA-MNPs may be due to the incorporation of drugs within the cavities LBA-MNPs [61]. A decrease in size occurs in the case of HES-CA-MNPs in contrast with CA-MNPs and may be due to a decrease in aggregation, as MNPs tend to aggregate rapidly due to magnetic dipole [62]. The PDI suggests the uniform dispersion of nanosuspension; a PDI value of more than 0.5 indicates the size broadening of NPs [63]. The PDI values of CA-MNPs, LBA-MNPs, HES-CA-MNPs, CUR-LBA-MNPs, and AmpB-LBA-MNPs are represented in Table 2. The experimental PDI value revealed that the drug-loaded formulation has more uniform colloidal dispersibility in comparison to unloaded analogs, suggesting higher colloidal stability of nanoformulations. Nanoparticle-based formulations are increasingly utilized for site-specific delivery. Literature analysis showed that nanoparticles less than 1000 nm can easily permeate the biological barriers to transport the drug at the desired site of action in increased amounts [64]. Nanoparticles had nearly spherical morphology regardless of drug inclusion, which shows the stability of nanostructures, as shown by AFM (Figure 3), consolidating the findings of our study.

### 3.4. Drug-Loading Efficiency 

The loading capacity and controlled release of drugs are generally related to the chemical nature of the drug and the nature of interaction with the carriers [65]. HES is a weakly acidic hydrophobic molecule containing a phenolic skeleton. The entrapment efficiency of HES within CA-MNPs was found to be 76.3 ± 2.45%. The significant adsorption of HES may be attributed to the increased surface hydrophobicity in the form of CA onto the surface of MNPs. Furthermore, it was shown through FTIR that CEF involves in chelation with MNPs, which is another factor for higher drug absorption [34]. In the case of CUR-LBA-MNPs and AmpB-LBA-MNPs, the loading efficiency was found to be 43 ± 5.4% and 80.1 ± 1.32%, respectively. The higher amount of loading may be attributed to the hydrophobic cavities and increased secondary interaction in the form of LBA moiety [34], which favors the encapsulation of hydrophobic drugs [66]. 

### 3.5. Drugs and Drug–Nanoparticle Formulations Presented Imperative Bactericidal Activities against MDR Pathogenic Bacteria

**Figure 4:**
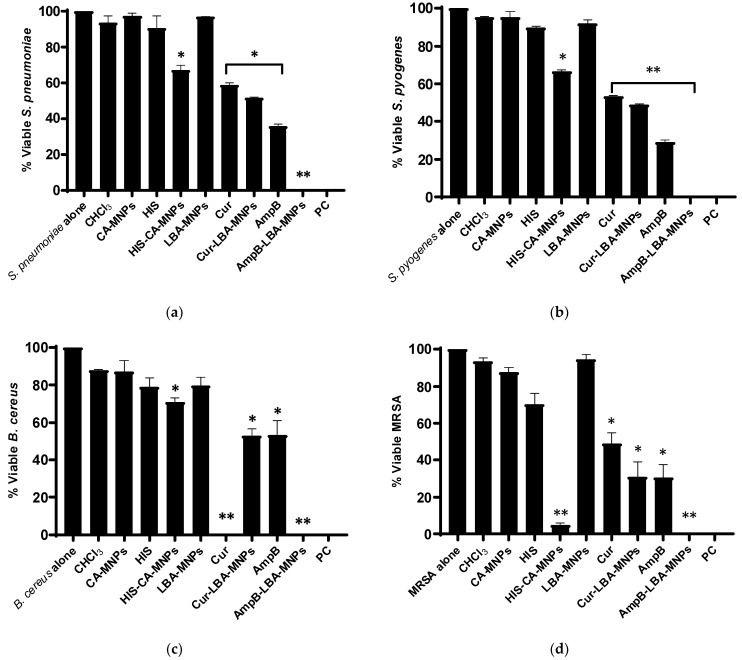

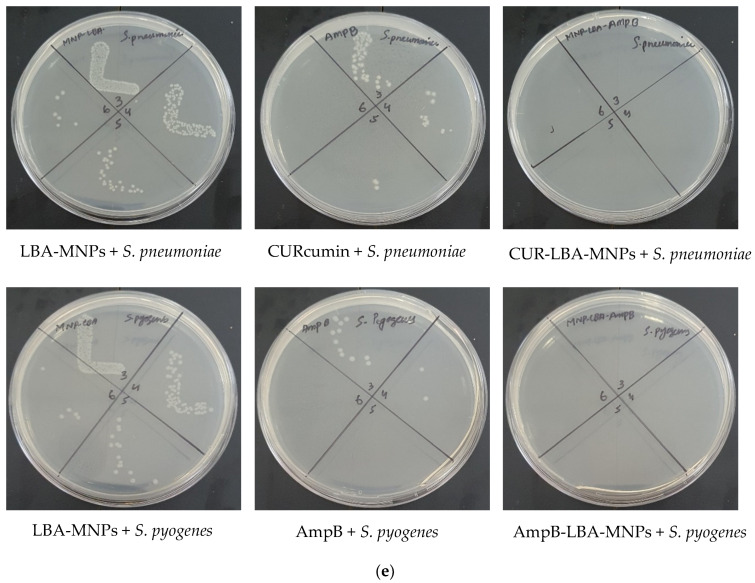
Drugs and drug-based magnetic NPs presented important antibacterial activity against Gram-positive pathogenic bacteria. Briefly, bacteria (1 × 106) were incubated with different drugs conjugated with MNPs and nanoparticles alone for 2 h at 37 °C. Next, the cultures were ten-fold serially diluted and plated onto the nutrient agar plates, and the plates were incubated overnight at 37 °C and counted the viable bacterial colonies on the following day. For negative control, bacteria were incubated in PBS alone, whereas for positive control, gentamicin (100 μg/mL) was used. (**a**) Antibacterial effects against *S. pneumonae*; (**b**) bactericidal activity against *S. pyogenes*; (**c**) against B. cereus; (**d**) against MRSA; (**e**) illustrative antibacterial effects against *S. pneumonae* and *S. pyogenes*. The data are expressed as the mean ± standard error of several independent experiments performed in duplicate, where (*) and (**) represents when *p* ≤ 0.05 and *p* ≤ 0.01 respectively.

Drugs alone (HES, CURCUR, and AmpB), NPs alone, and their nanoformulations were assessed for their bactericidal effects. The overall results revealed that drugs and drug–NP formulations offered significant antibacterial against Gram-positive MDR bacterial isolates (Figure 4a–e). Among all the Gram positive, HES-CA-MNPs presented important antibacterial activity against *S. pneumoniae* (33%) and *S. pyogenes*, reducing their viability up to 67% and 66%, respectively (*p* ≤ 0.05, using Student’s *t*-test, two-tailed distribution) (Figure 4a,b). Similarly, curcumin, CUR-LBA-MNPs, AmpB, and AmpB-LBA-MNPs showed notable bactericidal effects against these bacteria. Interestingly, AmpB-LBA-MNPs abolished 100% bacterial viability (Figure 4a,b). Similar patterns of antibacterial activities were found against *B. cereus* and MRSA (Figure 4c,d). Against *B. cereus*, both curcumin and AmpB-LBA-MNPs showed 100% bactericidal activity (Figure 4c), whereas HES-CA-MNPs and AmpB-LBA-MNPs eliminated 95% and 100% of bacterial growth against MRSA, respectively (Figure 4d). Some representative images of the bactericidal activities are shown in Figure 4e.

**Figure 5:**
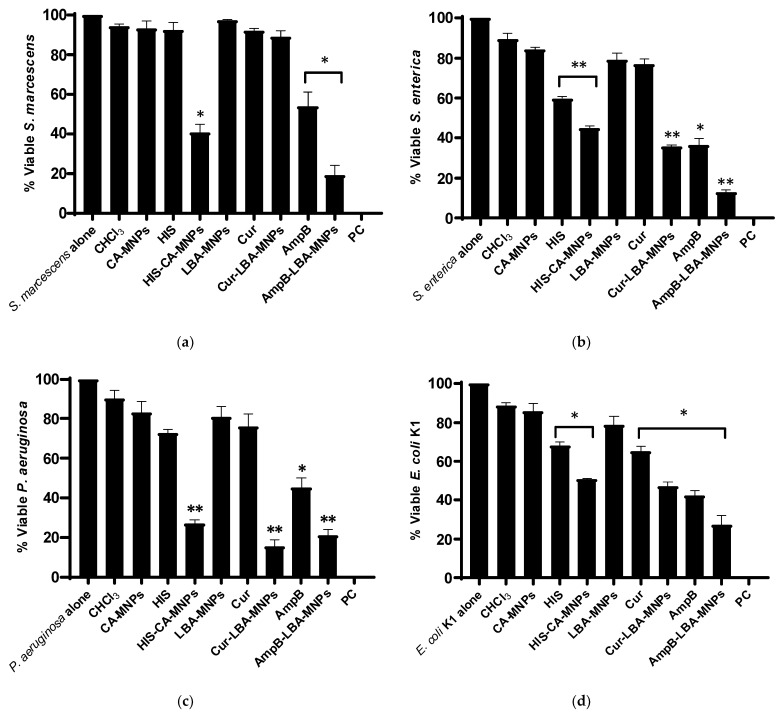

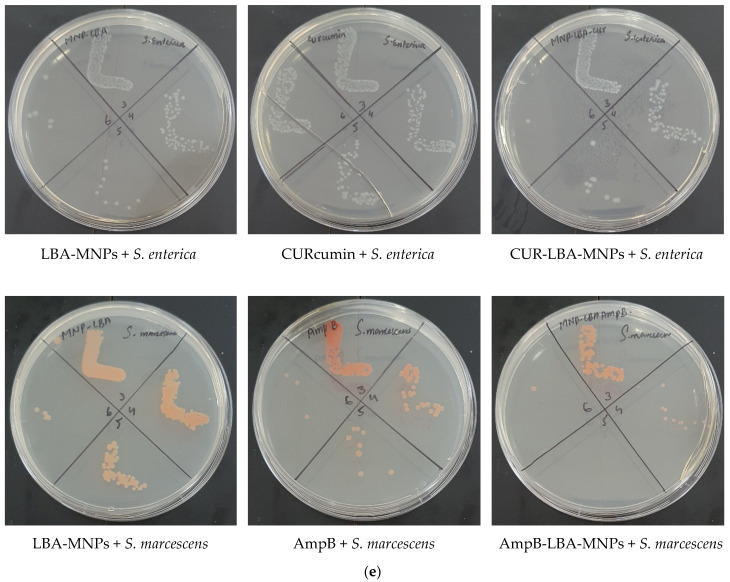
Drugs and drug-based magnetic NPs presented important antibacterial activity against Gram-positive pathogenic bacteria. Briefly, NPs and drug conjugates were incubated with one million bacteria at 37 °C for 2 h. After this, cultures were serially diluted (ten-fold) and plated on nutrient agar plates. The plates were incubated overnight at 37 °C, and subsequently, viable bacteria were counted. For controls, bacteria incubated alone in PBS and with gentamicin (100 μg/mL) were used as negative and positive controls, respectively. (**a**) Antibacterial effects against *S. marcescens*; (**b**) bactericidal activity against *S. enterica*; (**c**) against *P. aeruginosa*; (**d**) against *E. coli* K1; (**e**) illustrative antibacterial effects against *S. enterica* and *S. marcescens*. The data are expressed as the means ± standard error of several independent experiments performed in duplicate where (*) and (**) represents when *p* ≤ 0.05 and *p* ≤ 0.01 respectively.

Among Gram-negative bacteria, HES-CA-MNPs, AmpB, and AmpB-LBA-MNPs showed significant antibacterial activity against *S. marcescens* (*p* ≤ 0.05) (Figure 5a). AmpB-LBA-MNPs presented 81% of bactericidal activity against *S. marcescens.* Against *S. enterica*, HES reduced 60% of viability and was further reduced up to 45% when hesperidin was conjugated with cinnamic-acid-based magnetic NPs (*i.e.*, HES-CA-MNPs) (Figure 5b). curcumin alone did not show antibacterial activity but upon conjugation with lactobionic-acid-based MNPs significantly eliminated 65% of *S. enterica* (*p* ≤ 0.01) (Figure 5b). Similarly, AmpB and AmpB-LBA-MNPs abolished 63% and 87% of bacteria. HES-CA-MNPs showed promising antibacterial activity against *P. aeruginosa,* while the hesperidin alone failed to show any effects (Figure 5c). Similarly, curcumin alone had no effects, but after loading onto LBA-MNPs, the bactericidal effects were found significant, i.e., 85% (*p* ≤ 0.01) (Figure 5c). Both AmpB and AmpB-LBA-MNPs exhibited important antibacterial properties, but the cidal effects of AmpB were further enhanced after conjugation with LBA-MNPs. In the case of *E. coli* K1, all the drugs and drug–NP counterparts presented remarkable antibacterial effects except the MNPs alone (i.e., CA-MNPs and LBA-MNPs) (Figure 5d).

The MIC values of Hesperidin, curcumin, Amphotericin B, and their conjugated NPs against MRSA and *P. aeruginosa* are summarized in Table 3. The overall findings revealed that drugs and drug-loaded NPs exhibited substantial bactericidal properties against the MDR clinical isolates.

### 3.6. Drugs and Drug–NP Conjugates Confirmed Marginal Cytotoxicity

**Figure 6:**
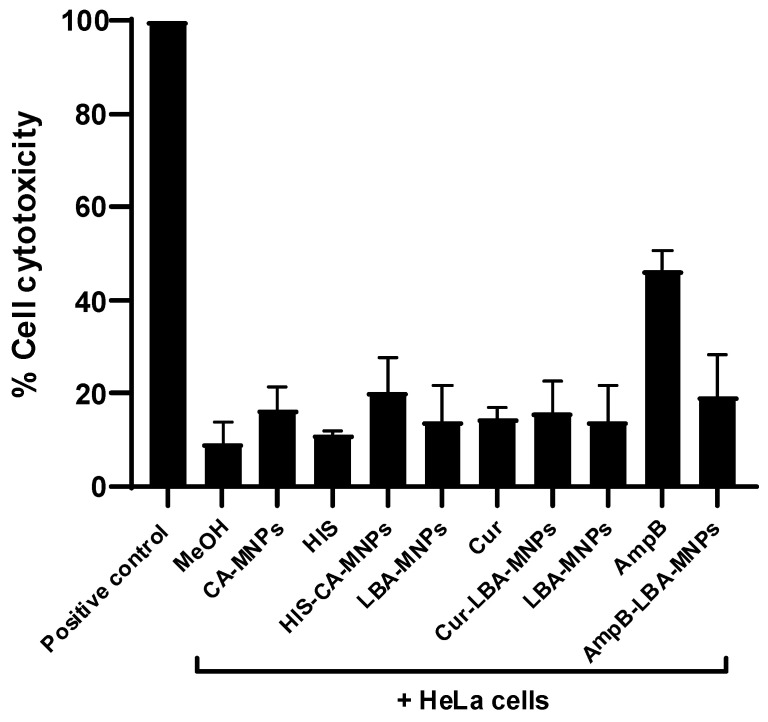
Drugs and drug-loaded MNPs revealed negligible cytotoxic effects against HeLa cell lines. Human cells were grown in 96-well plates up to 80–90% confluency, as discussed in Materials and Methods. Next, cell monolayer was treated with NPs, drugs, and drug–NP combinations for 24 h at 37 °C in 95% humidity and 5% CO_2_. Cells alone in RPMI were taken as negative control. The data are presented as the mean ± standard error of several independent experiments performed in duplicates. Data were analyzed using Graph Pad Prism software (8.0.2).

Results from the LDH assays revealed all the drugs, MNPs and drug–MNPs showed marginal cytotoxic effects against human cells (Figure 6). AmpB alone showed 46% cytotoxicity against HeLa cells when compared to positive control (100%). 

### 3.7. In Vitro Release Study 

**Figure 7:**
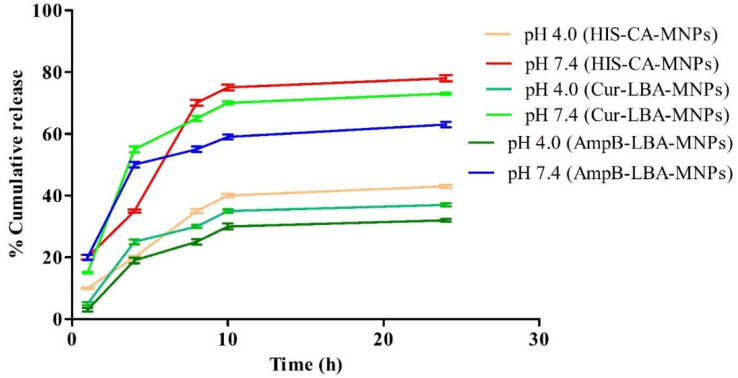
In vitro release profiling of HES, CUR, and AmpB from CA-MNPs and LBA-MNPs nanostructures at different pH.

Slightly acidic conditions (pH 4.0) and blood physiological conditions (pH 7.4) were used to evaluate the in vitro release profile of HES form CA-MNPs, CUR form LBA-MNPs, and AmpB form LBA-MNPs (Figure 7). The maximum drug release in case of HES-CA-MNPs of 35 ± 0.4% at pH 7.4 and 15 ± 0.2% at pH 7.4 was observed after 8 h and then persisted up to 24 h. In the case of CUR-LBA-MNPs, the maximum release of 20 ± 0.5% at pH 4.0 and 40 ± 0.9% at pH 7.4 was observed after 4 h and then sustained for 24 h (Figure 7). AmpB-LBA-MNPs showed a maximum release of 17 ± 0.7% at pH 4.0 and 30 ± 0.9% at pH 7.4 after 4 h and then persisted for 24 h. The outcomes suggest the stability of HES-CA-MNPs, CUR-LBA-MNPs, and AmpB-LBA-MNPs in acidic conditions, potentially suggesting that similar persistence might happen in the acidic environment of the stomach [15].

## 4. Discussion

Antibiotic resistance is on the rise around the world, threatening to undo the progress that has been made in treating bacterial infectious diseases [67]. For most low- and middle-income countries, inaccessibility to antibiotics remains a major challenge. Pneumonia alone kills about 1 million children under the age of five every year, and an estimated 445,000 could be prevented if medications for community-acquired pneumococcal infections were universally available [68]. Multidrug resistance in human bacterial pathogens has threatened the clinical effectiveness of the existing antibiotics, which directed the discovery of new drugs [69]. Antibiotic resistance, the decisive cause of elevated morbidity and mortality rates as well as increased treatment costs, is considered one of the major global public health threats [70,71]. Increased resistance against currently available drugs has also been observed in species of bacterial family *Enterobacteriaceae,* which contains important human and animal pathogens, including *Salmonella* and *Escherichia coli* [72]. From this perspective, it is evident that there is a pressing need to identify and develop innovative antibiotics, as well as unique antimicrobial treatments that could potentially work in tandem with traditional chemotherapy. The use of nanoparticles (NPs) could be a promising technique for treating MDR infections [73,74,75,76]. Because of their unique physical and chemical properties, NPs have demonstrated therapeutic potential in this regard [77,78,79]. Antibacterial nanoparticles can target several biomolecules, potentially reducing or preventing the spread of MDR bacteria [80]. For example, in our recent study, ZnO NPs conjugated with clinically approved drugs exhibited promising antibacterial effects against several MDR bacteria [43]. Similarly, magnetic iron oxide and mesoporous silica nanoparticles showed significant bactericidal properties against clinical MDR bacteria [34]. Flavonoids-based green synthesized gums-stabilized nanoparticles effectively eradicated Gram-positive and Gram-negative MDR species [22]. In the present work, magnetic NPs were synthesized and then conjugated with different drugs (HES, CURCUR, and AmpB). The NPs and drug–NP formulations were characterized using atomic force microscopy, Fourier transform-infrared spectroscopy, and Zetasizer and then tested for antibacterial strength against several multidrug-resistant Gram-positive bacteria. The results have shown that upon conjugation, the antibacterial activity of NPs as well as drugs alone was significantly enhanced against pathogenic bacteria. Interestingly, the NPs and drug–NP nanoconjugates except AmpB presented minimal cytotoxicity towards human cell lines.

Among all the NPs, drug–NP conjugates AmpB-LBA-MNPs showed consistent and most promising antibacterial activity against both the Gram-positive and Gram-negative bacteria. This activity was more robust against Gram-positive isolates, abolishing 100% bacterial viability upon 24 h incubation. AmpB is a potent antifungal drug [81] and has been shown to have weak or no antibacterial properties, although the derivatives of AmpB have been found to have some in vitro antiviral activity against the human immunodeficiency virus [82]. Among Gram-negative bacteria, AmpB-LBA-MNPs showed the highest bactericidal activity against *S. enterica* killing 87% bacterial population. Similarly, hesperidin alone failed to hamper bacterial growth except against *S. enterica* and *E. coli* K1 (showed bactericidal effects); however, after conjugating with CA-MNPs, the antibacterial properties enhanced significantly exerted potent bactericidal activity against all tested clinical isolates. This is because the functionalized cinnamic has inherent antibacterial properties, and upon drug loading onto nanoparticles, the synergistic antibacterial efficacy was also observed; the drug-loaded nanoformulations have been demonstrated to have a number of advantages over traditional administration and delivery methods, such as the potential to transport drugs to a specific region, i.e., intracellular infection [83,84]. NPs can also be used to facilitate sustained drug release, reducing dose regimes [85]. Furthermore, nanoparticles can conceal the encapsulated drug, minimizing systemic cytotoxic effects caused by traditional free drug administration methods [86]. Hesperidin-loaded green synthesized gums-stabilized nanoparticles exhibited notable antibacterial properties against MDR bacteria [22]. Hesperidin-loaded PLGA NPs showed promising antibacterial effects against *Escherichia coli*, *Enterobacter aerogenes*, *Klebsiella pneumoniae,* and *Pseudomonas aeruginosa* [87]. Somu et al. (2021) reported the antimicrobial and antioxidant activity of curcumin and further revealed that upon conjugation with self-assembled lysozyme nanoparticles, these activities were significantly improved [88]. In another study, Shanmugam et al. (2021) revealed that the addition of AgNPs with the curcumin-assisted chitosan nanocomposites showed notable antibacterial effects against *S. aureus* and *P. aeruginosa* [89]. In the present study, enhanced antibacterial activities were observed against both the Gram-negative and Gram-positive MDR bacteria. 

The drugs and drug-loaded nanoconjugates were further tested for their antibacterial activity at graduate concentrations to evaluate their MIC values. Interestingly, AmpB after conjugation, i.e., AmpB-LBA-MNPs, indicated MIC values at 25 µg/mL against MRSA, while HES-CA-MNPs showed MIC at 105 µg/mL. Against *P. aeruginosa*, CUR-LBA-MNPs revealed MIC at 115.6 µg/mL. The MIC values demonstrated by hesperidin were 1.13 mg mL^−1^, 1.27 mg mL^−1^, 1.33 mg mL^−1^, and 1.53 mg mL^−1^ against *E. coli*, *P. aeruginosa*, *B. cereus,* and *S. aureus,* respectively. Additionally, the interaction of sodium nitrite and hesperidin showed strong synergic effects on *B. cereus* and *P. aeruginosa* [45]. Similarly, curcumin was found to exhibit MIC at 0.438 mg/mL, which was significantly decreased to 0.114 mg/mL when combined with polysaccharide nanoparticles against *Staphylococcus mutans* [46]. 

Finally, the drugs and drug-loaded MNPs presented minimal cytotoxicity against human cell lines. Human cells treated with varying doses of curcumin nanoparticles demonstrated a greatly improved percentage of cell viability against baby hamster kidney (BHK) normal cell lines [90]. Recently, Saeed et al. (2022) reported that CURCUR and CURCUR-loaded nanovesicles had low cytotoxic effects on human keratinocytes [28]. HES-loaded gum acacia-based NPs showed negligible cytotoxic effects against human cell lines [22]. AmpB loaded with metronidazole conjugated magnetic nanoparticles produced minimal cytotoxic effects against human cell lines [49].

According to the results obtained from the present study, drugs and drug-loaded MNPs exerted potent bactericidal activity against Gram-positive and Gram-negative MDR isolates. These nanoconjugates revealed MIC at lower concentrations (i.e., micrograms). AmpB-LBA-MNPs showed consistent and potent antibacterial properties against all the tested MDR bacteria. The results were stronger against Gram-positive bacteria. Moreover, the drugs and drug-loaded nanoformulations displayed higher biocompatibility with human cells. Further intensive ex vivo and in vivo research are needed to develop an NPs-based drug formulation that could be applied topically or delivered systemically to treat deep tissue and systemic bacterial infections. Our findings suggest that combining the unique features of multiple nanomaterials in a synergistic fashion could be a good strategy for preventing and treating bacterial infections.

## Figures and Tables

**Table 1:** Bacteria used in the study.

Bacteria	Strain
*Bacillus cereus*	MTCC 131621
Methicillin-resistant *Staphylococcus aureus*	MTCC 381123
*Streptococcus pneumoniae*	ATCC 13883
*Streptococcus pyogenes*	ATCC 49399
*Salmonella enterica*	ATTC 14028
*Escherichia coli* K1	MTCC 710859
*Serratia marcescens*	MTTC 13880
*Pseudomonas aeruginosa*	ATCC 10145

**Table 2:** Average size and PDI of drug-loaded CA-MNPs, LBA-MNPs, HES-CA-MNPs, CUR-LBA-MNPs, and AmpB-LBA-MNPs nanocomposites.

Nanoparticles	Average Size (nm)	PDI
CA-MNPs	300 ± 20.1	0.39 ± 0.050
HES-CA-MNPs	243.4 ± 25.3	0.24 ± 0.010
LBA-MNPs	153.8 ± 10.4	0.30 ± 0.018
CUR-LBA-MNPs	189.2 ± 5.7	0.18 ± 0.024
AmpB-LBA-MNPs	175.3 ± 13.5	0.29 ± 0.053

**Table 3:** Minimum inhibitory concentration (MIC) of drugs and drug-loaded NPs (μg/mL).

Drugs/Formulations	*P. aeruginosa*	MRSA
	MIC	MIC
HES	357.1	333.3
HES-CA-MNPs	136.9	105.25
Curcumin	416.6	175.5
CUR-LBA-MNPs	115.6	130.25
AmpB	180.8	141.85
AmpB-LBA-MNPs	122.5	25

## Data Availability

The data presented in this study are available on request from the corresponding author.
